# Percutaneous radiofrequency ablation for osteoid osteoma: How we do it

**DOI:** 10.4103/0971-3026.44523

**Published:** 2009-02

**Authors:** Bhavin Jankharia, Nishigandha Burute

**Affiliations:** Radiologists, Piramal Diagnostics - Jankharia Imaging

**Keywords:** Osteoid osteomas, radiofrequency ablation

## Abstract

**Aims and Objectives:**

To describe our technique for performing radiofrequency ablation (RFA) in osteoid osteoma and to evaluate the results of treatment.

**Materials and Methods:**

We evaluated 40 patients in whom RFA was performed for osteoid osteomas between October 2005 and February 2008. The lesions were located in the femur (n = 22), tibia (n = 10), humerus (n = 2), acetabulum (n = 2), radius (n = 1), fibula (n = 1), patella (n = 1), and calcaneum (n = 1). The procedure was performed using a standard technique.

**Results:**

Technical success was achieved in all patients, with intranidal localization of the needle and complete ablation. All patients were fully weight bearing 2–3 h after the procedure. Successful pain relief was achieved in all patients within 48 h. Immediate complications included a case of minor thermal skin burn and a small cortical chip fracture, which healed on its own. There were no delayed complications. The average follow-up period was 12 months. Two patients (5% of cases) had recurrence of pain after intervals of 5 and 8 months, respectively, following the ablation; this was due to recurrence of the lesion. Complete pain relief was however achieved after a second ablation in both cases. Thus, our primary and secondary clinical success rates were 95 and 100%, respectively.

**Conclusion:**

RFA is a safe, quick, minimally invasive, and extremely effective method for the management of osteoid osteomas.

## Introduction

Osteoid osteoma is an extremely painful benign skeletal tumor seen in young individuals. Anti-inflammatory medications have been traditional used for pain management. Although surgery is the definitive treatment, difficulty in lesion localization and the need for extensive dissection pose a problem. Radiofrequency ablation (RFA) has been found to be a safe, fast, and reliable method of treating osteoid osteomas.[1–3] We would like to present our work on 40 patients with osteoid osteomas in whom we successfully performed RFA. We also describe in detail our method of performing this procedure.

## Materials and Methods

During the period from October 2005 to February 2008, 40 patients underwent percutaneous RFA for osteoid osteomas. The lesions were located in the femur (*n* = 22), tibia (*n* = 10), humerus (*n* = 2), acetabulum (*n* = 2), radius (*n* = 1), fibula (*n* = 1), patella (*n* = 1), and calcaneum (*n* = 1). One patient had had percutaneous RFA 2 years ago at another center and presented with recurrence of symptoms.

### Criteria for diagnosis of osteoid osteoma

**Figure 1:**
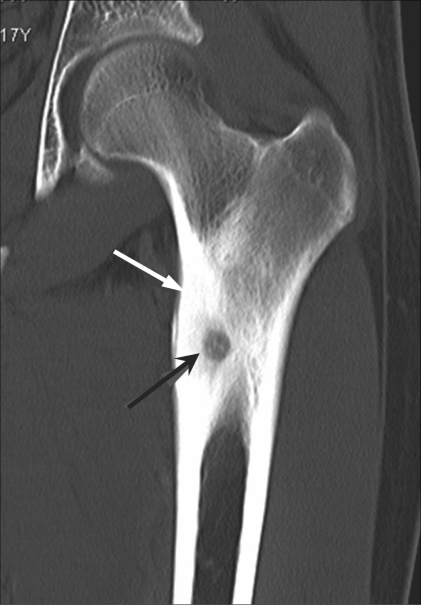
Osteoid osteoma of the femur. Coronal CT scan shows a radiolucent nidus (black arrow) with surrounding bony sclerosis and cortical thickening (white arrow)

We diagnosed osteoid osteomas on the basis of the following:

- Presence of pain that was worse at night and relieved by administration of oral anti-inflammatory medication
- Documentation of a radiolucent nidus with surrounding bony sclerosis and cortical thickening on CT images [Figure 1].

### Preprocedure

The patients and parents were given detailed explanations about the procedure and the surgical and medical alternatives available; informed written consent was obtained in all cases. Before the procedure, we confirmed that prothrombin time and international normalized ratio (INR) were normal. An anesthetist's evaluation was also carried out. Prophylactic antibiotic (cefotaxime 1 gm) and atropine were administered immediately before the procedure.

**Figure 2:**
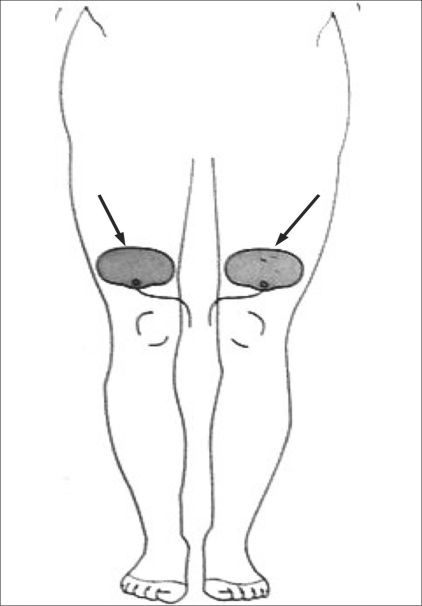
Frontal diagram of the lower limbs show the method of placement of the dispersive grounding pads (arrow)

Dispersive grounding pads were applied either on the patient's thighs or on the back, in proper alignment with each other and with good skin contact; both pads were placed at approximately equal distance from the site of ablation and as close to the ablation site as possible so as to allow the shortest current path through the patient [Figure 2]. Towels were placed between the trunk and the patient's arms on either side, as well as between the legs, to reduce the risk of skin–skin contact burns.

Deep sedation was induced with ketamine or another appropriate anesthetic.

**Figure 3 (A–C):**
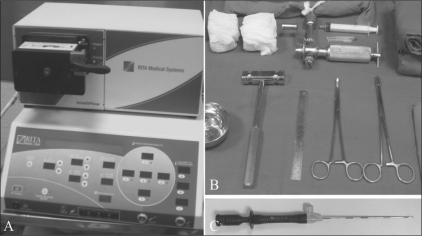
RFA equipment. The generator (A), the set required for performing the procedure (B), and the electrode (C) are shown

The RFA equipment comprised an 11-guage diamond-tip bone biopsy needle (Cook Inc, USA), a K-wire, a hammer and drill, a 6-cm bevel-tip introducer (RITA Medical Solutions, USA), and a standard 1.2-cm three-pronged Starburst SD (RITA Medical Solutions, USA) electrode [Figures 3a–c].

### Lesion localization

The lesions were localized with the help of a 64-slice multidetector-row CT scanner (Somatom 64, Siemens, Erlangen, Germany), with multiplanar evaluation to confirm accurate needle position within the nidus. In three patients, CT fluoroscopy was used to facilitate rapid and accurate needle localization.

### Procedure

**Figure 4:**
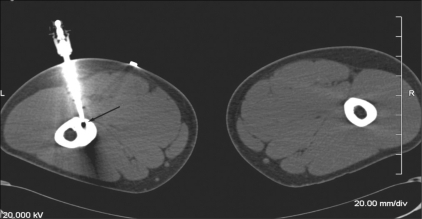
Osteoid osteoma of the femur. CT scan in the supine position shows the position of the electrode (arrow) within the nidus

**Figure 5:**
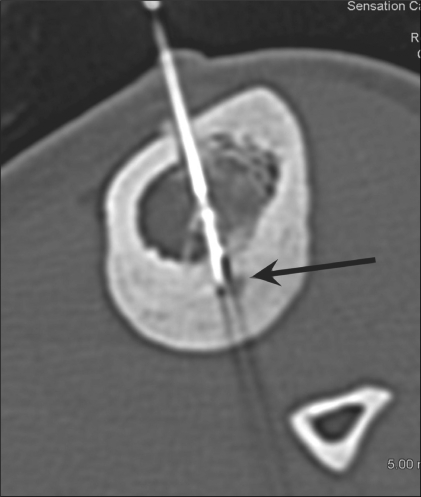
Osteoid osteoma of the tibia. CT scan in the supine position shows an approach to the lesion in the posterior cortex, through the nonaffected anterior cortex (arrow)

After skin preparation and proper sterilization, local anesthesia was administered. The position of the lesion and ease of access and the relationship with adjacent neurovascular structures were assessed [Figure 4]. To minimize the possibility of thermal burns, the tip of the probe was inserted deep so that it did not lie near the skin surface. An approach through the nonaffected opposite cortex was employed in one case [Figure 5]. An 11-guage bone biopsy needle was introduced into the lesion under CT guidance. In cases where there was marked perilesional sclerosis, the tumor was reached through a drill advanced over an appropriate Kirchner guidewire. Under aseptic precautions, a 12-cm long, 14-gauge side-deployment electrode was then introduced into the osteoid osteoma nidus through a coaxial system. The electrode was connected to the RF generator (RITA Medical Solutions, USA) and the tip temperature was increased to 90°C. RFA was performed at 90°C for a minimum of 5 min. After the procedure, a small pressure dressing was applied at the percutaneous puncture site.

### Postprocedure

The average time for sedation to wear off was 2–3 h. Painkillers were administered as necessary. Patients with tumors in the legs (weight-bearing sites) were advised to avoid vigorous activities and sports such as jumping or long-distance running for 1 month. Other activities were not restricted.

## Follow-up

The mean follow-up period was 8 months (range 2–12 months). Follow-up assessment was through telephonic enquiry (clinical follow-up) as well as radiological evaluation for regression of the lesion over a period of time.

### Clinical follow-up

Telephonic enquiry was carried out every 24 h for 3 days and every week for 1 month for the assessment and confirmation of pain relief. There was often some pain on the day of the procedure; however, most patients affirmed that there was complete disappearance of pain within 48–72 h.

### Radiological follow-up

This comprised one MRI within 24–48 h after the procedure and repeat CT and MRI after 1 month and 1 year.

**Figure 6:**
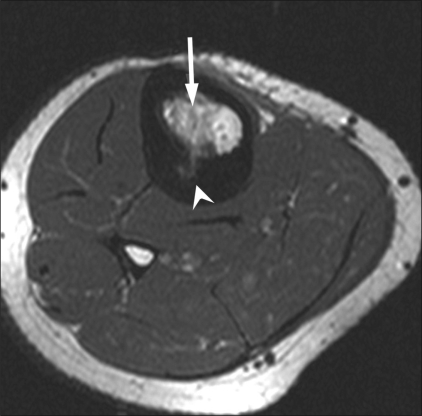
Osteoid osteoma of the tibia. Postprocedure T1W axial MRI shows the zone of ablation (arrow) surrounding the ablated nidus (arrowhead)

**Figure 7 (A, B):**
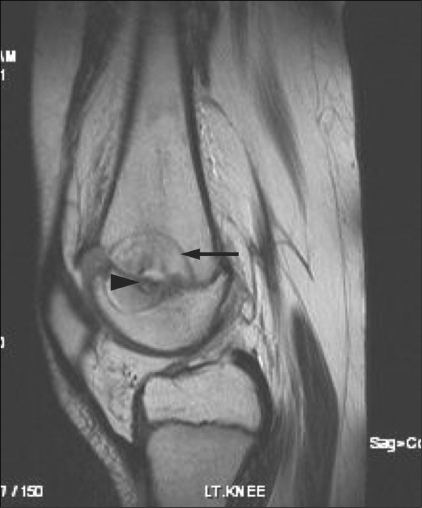
Follow-up MRI after RFA of a subphyseal femoral osteoid osteoma of the femur. A sagittal T1W MRI (A) shows an area of coagulation necrosis (arrow) surrounding the nidus (arrowhead). A coronal contrast-enhanced T1W MRI with fat suppression (B) shows a hypointense perfusion defect (arrow) surrounded by a thin enhancing rim (arrowhead)

Early findings on imaging that suggested a successful procedure included the presence of an ablation tract and a ‘zone of ablation’ (due to coagulation necrosis) surrounding the tumor nidus on MRI [Figure 6]. Expected late findings on follow-up MRI at 1 year included sclerosis and regression of the lesion and reduction of the bone edema, with a well-demarcated low-signal zone of coagulation necrosis around the tumor nidus [Figure 7A]. After a successful procedure, contrast images showed a perfusion defect surrounded by a thin enhancing rim. [Figure 7B]. Follow-up CT scan did not reveal a significant change in the trabecular density.

### Duration of the procedure

The duration of the procedure (from the time the patient entered the CT room and including the time taken for anesthesia) was approximately 90 min.

### Criteria for assessment of success of the procedure

The procedure was considered technically successful if the tip of the electrode was correctly placed into the nidus and the lesion was heated for the required time, maintaining the correct parameters. Clinical success was defined as complete relief from pain and return to normal activities.

A major complication was defined as an untoward event requiring prolonged hospitalization or treatment unrelated to the disease process. This would include a fracture or large skin burn requiring grafting. A minor complication was defined as a self-limiting sequela of the procedure, such as transient parasthesia or erythema or a small superficial skin burn adjacent to the RF probe or grounding pad.

## Results

Technical success was achieved in 100% of the cases, with correct intranidal localization of the needle and complete ablation. Immediate clinical success was observed in all. Two patients experienced recurrence of pain at 5 and 8 months, respectively, following the ablation. Complete pain relief was however achieved in both cases after a second ablation. Thus, our primary and secondary clinical success rates were 95% and 100%, respectively.

One 9-year-old boy had an intramedullary lesion in the distal right femur at the physeal plate. A caudal angulated approach was used to minimize the ablation injury to the physeal plate. Follow-up MRI revealed a well-defined semicircular zone of ablation on either side of the physeal plate.

### Complications

One patent developed a localized skin burn of about 6-mm diameter at the site of insertion of the RF probe. The lesion was located along the anterior border of the diaphysis of the humerus, 1.7 cm from the skin surface. The burn healed with local wound care.

Another patient developed a small chip fracture at the entry site of the needle into the lesion. However, since the cortex was thickened due to sclerosis, the fracture was clinically insignificant. The patient was advised to avoid strenuous physical activity and to avoid weight bearing for 6 weeks.

None of the other patients had any complications.

## Discussion

An osteoid osteoma is a benign skeletal tumor, usually less than 1.5 cm in diameter.[4] It is composed of woven bone and osteoid and is more commonly located in the appendicular skeleton.[5] There is a male preponderance, with a male: female ratio of 3:1.[3,4] It is commonly seen in children and young adults.[6]

The disease commonly presents with focal bone pain at the tumor site in a child or a young adult. The pain worsens at night, increases with activity, and is relieved by small doses of anti-inflammatory medication. The pain is presumed to be a result of local vasodilatation resulting from elevated levels of PGE_2_ at the site of the tumor.[7] Spinal osteoid osteomas may, in addition, lead to scoliosis.[8] If the tumor is near the growing end of a bone in a child, increased blood flow may cause deformities in the form of either overgrowth or undergrowth of the limb.[8]

These tumors usually regress spontaneously, the mechanism probably being bone infarction.[9]

### Imaging Radiography and CT scan

**Figure 8:**
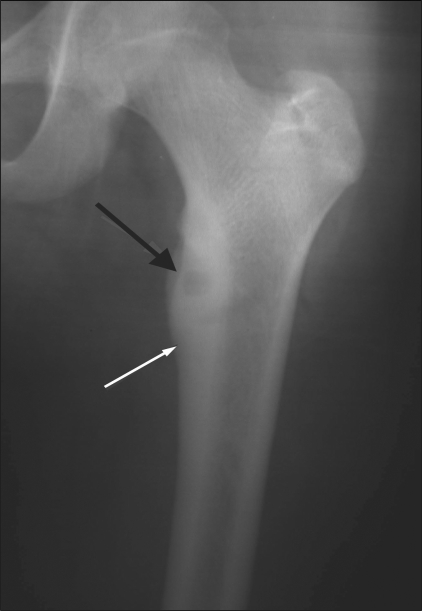
Osteoid osteoma. Plain radiograph shows a typical metadiaphyseal cortical osteoid osteoma involving the upper end of the femur. The nidus is well seen (black arrow) along with the surrounding cortical thickening (white arrow)

Osteoid osteomas show a central circular or oval lucent nidus, with surrounding cortical and endosteal sclerosis. Subperiosteal and cortical tumors show considerable sclerosis, whereas endosteal tumors show a negligible amount. Sclerosis may be totally absent in subarticular and intracapsular tumors. Some of these lesions also exhibit a periosteal reaction. The size of the lesion (the nidus) usually varies from 0.5 to 2 cm [Figure 8].

**Figure 9:**
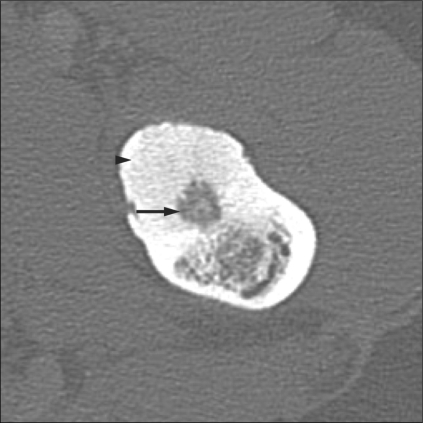
Osteoid osteoma in the femur. Axial CT scan shows a nidus (arrow) within the cortex, with surrounding dense sclerosis (arrowhead)

CT scan is the gold standard for diagnosis. It precisely shows the nidus, which may have variable degrees of amorphous, punctate, or dense mineralization within [Figure 9].

### MRI

**Figure 10:**
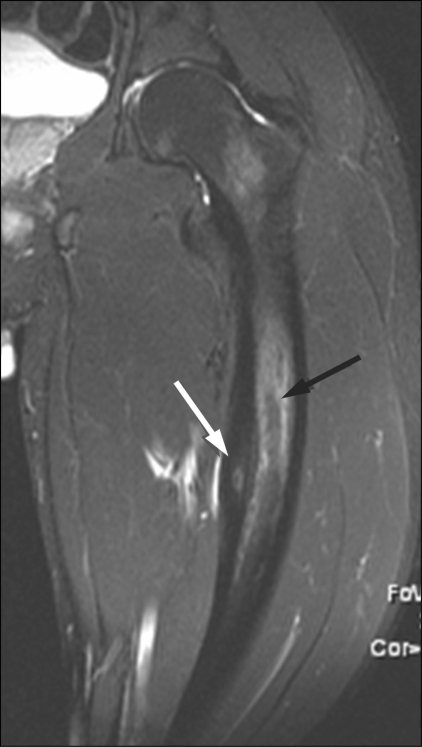
Osteoid osteoma. STIR coronal MRI shows the nidus (white arrow) along with marrow edema (black arrow)

The nidus is isointense to muscle on T1W and slightly hyperintense on T2W sequences. Bone marrow edema around the nidus is present in approximately 60% of patients [Figure 10]. Dynamic contrast-enhanced MRI shows rapid uptake in the lesion during the arterial phase followed by early partial washout, as against the slower, progressive enhancement of the surrounding marrow.[10] When CT scan is equivocal, the presence of rapid initial uptake on dynamic contrast-enhanced MRI helps in confirming the diagnosis.

Soft-tissue edema may be seen adjacent to the tumor.[11] Contrast-enhanced T1W MRI has been shown to be the most accurate technique to demonstrate the zone of coagulated tissue following treatment.[12]

### Radionuclide imaging

Though single-photon emission computed tomography (SPECT) with Tc99m diphosphonate shows activity at the tumor site, along with the double-density sign,[13] the usefulness of this technique is debatable.

### Surgery

Surgery was the only treatment available for osteoid osteomas until recently.[8] Difficulty in lesion localization during surgery is a major problem.[1,3,8] Moreover, successful surgery necessitates complete removal of the tumor and thus extensive resection; this causes tissue damage and may lead to structural weakening.[1,3,8] Surgery also carries a high risk of complications and requires a long recovery period.[3,8] Surgery for tumors in weight-bearing areas also entails a long period of limited weight bearing.[8]

Arthroscopic removal has been attempted for intra-articular lesions.[14]

### Interventional techniques

Several techniques are available for osteoid osteoma ablation. The most accepted method is percutaneous ablation by using RF waves. Percutaneous resection with ethanol ablation[15] and laser ablation,[16] have also been tried.

Percutaneous RFA is a widely used interventional technique. It allows the precise delivery of heat under image guidance to the targeted tissue. High-frequency alternating current at 500,000 Hz transmitted through a delivery probe induces local ionic agitation and frictional heat in the tissue about the probe, leading to coagulation necrosis.[17]

**Figure 11:**
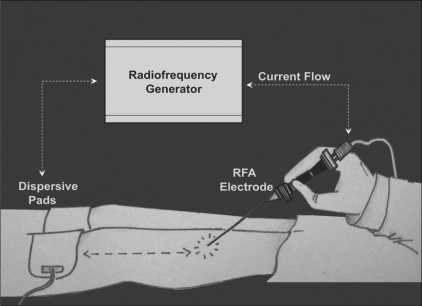
This diagram shows the method of performing an RFA

RFA is usually performed under general or spinal anesthesia. We performed RFA using deep sedation and local anesthesia. The procedure described above is similar to the procedure described by other authors.[2,3] RFA is preferred because it does not necessitate hospitalization, it is not associated with significant complications, and it requires only a short convalescence period[18] [Figure 11]. RFA has limitations in the case of spinal lesions, and minimally invasive surgery (for preserving the structures) may be the better option in these cases.[19]

Percutaneous thermocoagulation under CT guidance has been performed to achieve ablation of spinal osteoid osteomas.[2] Laser thermal therapy[16] and percutaneous resection after intranidal ethanol administration[15] have also been attempted.

## Conclusions

Difficulty in lesion localization, the consequences of extensive dissection, the need for prolonged recuperation, as well as the risk of incomplete removal and therefore recurrence of the lesion, make surgery a less desired option in the management of osteoid osteomas. RFA, on the other hand, has proved to be a safe, quick, and minimally invasive method of management.

We have been able to achieve a high technical and clinical success rate, with minimal immediate and delayed complications and morbidity. Percutaneous RFA should be the method of choice for treating extraspinal osteoid osteomas.
